# Deregulation of oxidative phosphorylation pathways in embryos derived in vitro from prepubertal and pubertal heifers based on whole-transcriptome sequencing

**DOI:** 10.1186/s12864-024-10532-7

**Published:** 2024-06-24

**Authors:** Milena Traut, Ilona Kowalczyk-Zieba, Dorota Boruszewska, Joanna Jaworska, Sandra Gąsiorowska, Krzysztof Lukaszuk, Katarzyna Ropka-Molik, Katarzyna Piórkowska, Tomasz Szmatoła, Izabela Woclawek-Potocka

**Affiliations:** 1 Department of Gamete and Embryo Biology, Institute of Animal Reproduction and Food Research, Polish Academy of Sciences, Olsztyn, 10-747 Poland; 2 Department of Obstetrics and Gynecology Nursing, Medical University of Gdansk, Gdansk, 80-210 Poland; 3 Invicta Research and Development Center, Sopot, 81-740 Poland; 4 Department of Animal Molecular Biology, National Research Institute of Animal Production, Krakowska 1 St, Balice, 32-083 Poland; 5 Center for Experimental and Innovative Medicine, University of Agriculture in Krakow, Redzina 1c, Krakow, 30-248 Poland

**Keywords:** Next-generation sequencing, Mitochondria, Blastocyst, Prepubertal heifer, Cow

## Abstract

**Background:**

Although, oocytes from prepubertal donors are known to be less developmentally competent than those from adult donors it does not restrain their ability to produce full-term pregnancies. The transcriptomic profile of embryos could be used as a predictor for embryo’s individual developmental competence. The aim of the study was to compare transcriptomic profile of blastocysts derived from prepubertal and pubertal heifers oocytes. Bovine cumulus-oocyte complexes (COCs) were obtained by ovum pick- up method from prepubertal and pubertal heifers. After in vitro maturation COCs were fertilized and cultured to the blastocyst stage. Total RNA was isolated from both groups of blastocysts and RNA-seq was performed. Gene ontology analysis was performed by DAVID (Database for Annotation, Visualization and Integrated Discovery).

**Results:**

A higher average blastocyst rate was obtained in the pubertal than in the pre-pubertal group. There were no differences in the quality of blastocysts between the examined groups. We identified 436 differentially expressed genes (DEGs) between blastocysts derived from researched groups, of which 247 DEGs were downregulated in blastocysts derived from pubertal compared to prepubertal heifers oocytes, and 189 DEGs were upregulated. The genes involved in mitochondrial function, including oxidative phosphorylation (OXPHOS) were found to be different in studied groups using Kyoto Encyclopedia of Genes (KEGG) pathway analysis and 8 of those DEGs were upregulated and 1 was downregulated in blastocysts derived from pubertal compared to prepubertal heifers oocytes. DEGs associated with mitochondrial function were found: ATP synthases (ATP5MF-ATP synthase membrane subunit f, ATP5PD- ATP synthase peripheral stalk subunit d, ATP12A- ATPase H+/K + transporting non-gastric alpha2 subunit), NADH dehydrogenases (NDUFS3- NADH: ubiquinone oxidoreductase subunit core subunit S3, NDUFA13- NADH: ubiquinone oxidoreductase subunit A13, NDUFA3- NADH: ubiquinone oxidoreductase subunit A3), cytochrome c oxidase (COX17), cytochrome c somatic (CYCS) and ubiquinol cytochrome c reductase core protein 1 (UQCRC1). We found lower number of apoptotic cells in blastocysts derived from oocytes collected from prepubertal than those obtained from pubertal donors.

**Conclusions:**

Despite decreased expression of genes associated with OXPHOS pathway in blastocysts from prepubertal heifers oocytes, the increased level of *ATP12A* together with the lower number of apoptotic cells in these blastocysts might support their survival after transfer.

**Supplementary Information:**

The online version contains supplementary material available at 10.1186/s12864-024-10532-7.

## Background

In vitro embryo production (IVP) in cattle demands obtaining embryos of decent developmental competence, which is a great challenge for assisted reproductive technology [1]. Although the IVP of embryos from prepubertal animals is even more challenging, this procedure provides many more possibilities for the cattle breeding industry due to the greater chance for faster genetic progress. However, during IVP procedure of embryos from prepubertal animals, the developmental potential of the collected oocytes is still the major limiting factor for embryonic developmental potential, resulting in the procurement of various amounts of transferable blastocysts and subsequent pregnancies [2, 3].

In every single cell of the body, including the oocyte, the major mechanism involved in energy metabolism is ATP production, which depends on the normal function of mitochondria and is significant for proper embryo developmental competence [4, 5]. Mitochondria, the powerhouses of the cells, are not only involved in cell death and homeostasis by supporting redox homeostasis and producing intermediate metabolites for the pathways of cell signaling and gene expression; their crucial role is ATP production throughout the process of oxidative phosphorylation (OXPHOS) [6]. Moreover, in cows, the enzymes involved in the Krebs cycle are also responsible for epigenetic remodeling, which occurs during embryonic genome activation (EGA) [7].

Bovine embryonic development occurs in accordance with specific metabolic patterns, among which the production of energy is mainly associated with pyruvate oxidation through oxidative phosphorylation. During this process, one molecule of glucose is transformed into two molecules of pyruvate to produce 2 molecules of ATP [8]. During OXPHOS, 33 molecules of ATP are produced by the electron transport chain in the mitochondrial membrane [9, 10]. In the cow, it has been demonstrated that during early embryonic development, an increasing function of energy acquired from glycolysis was correlated with compaction and blastulation [11, 12]. The remarkable increase in glucose metabolism during early embryo development was also reported by Gardner [13] to significantly increase embryo viability. Moreover, it is worth noting that the deficient ATP content was associated with fertilization failure and inability to achieve the blastocyst stage in cattle [5].

To the best of our knowledge, there are no data in the literature on global gene expression in embryos derived from oocytes from prepubertal and pubertal heifers or whether the impaired early embryonic development from oocytes collected from prepubertal heifers depends on different ATP production capacities in the mitochondria of these embryos. We also planned to gain the knowledge if the oxidative phosphorylation pathway of embryos derived from oocytes collected from prepubertal and pubertal heifers may be a good predictor for embryo’s developmental competence. In the current study we presume that there is a potential for selecting transferable embryos not only on the basis of morphological criteria, but also on the basis of candidate transcripts. The studies of Chitwood et al. [14] presented the first application of RNA-seq technology in single bovine embryos, Graf et al. [15] provided detailed insight into the timing of gene activation during early bovine embryo development and Jiang et al. [16] demonstrated the comprehensive examinations of gene expression in bovine oocytes and preimplantation embryos and taking above the studies presented the transcriptome analysis of whole bovine embryos. Moreover, the analysis of transcriptome profiles of bovine embryos derived in vivo Kues et al. [17], Gad et al. [18] Jiang et al. [16] and Kropp et al. [19] or in vitro Gad et al. [18], Graf et al. [15], Kropp et al. [19] and Wei et al. [20] allowed us to understand the regulatory mechanisms concerning EGA and metabolic demands before preimplantation development of bovine embryos. Previous studies also proved that the different environmental conditions of embryo culture in vitro influenced the transcriptomic profiles of the obtained embryos [1, 18]. Moreover, Gad et al. (2012) reported that the culture conditions during maturation and early embryo development also influenced the metabolic activity and gene expression patterns of bovine embryos [18]. Finally, Morin-Doré et al. (2017) demonstrated the transcriptome profile of blastocysts derived from prepubertal and pubertal heifers oocytes by microarray method, however we decided to use NGS sequencing due to its better potential to gain deeper insight into the transcriptome profile.

The purpose of this study was to examine whether different developmental competence of the embryos derived from prepubertal and pubertal animals is affected by the heterogeneity of their transcriptomic profiles. Moreover, the expression of molecular markers associated with mitochondrial function, expressed by the genes involved in OXPHOS, might be applied as a predictable marker for embryonic developmental capability. Thus, in this study, the leading objective was to identify and compare the transcriptional status of bovine blastocysts obtained in vitro from prepubertal and pubertal heifers. The presented study is the continuation of the previous research published in Theriogenology [21] which aimed to demonstrate the differences between the level of the mitochondrial DNA content and the expression of genes involved in mitochondrial function and embryo’s developmental competence in blastocysts derived from prepubertal and pubertal heifers. On the basis of the literature and our previous study [21], we presume that the regulatory gene pathways involved in the OXPHOS pathway in blastocysts derived from prepubertal versus pubertal heifers can reflect different developmental competence of the embryos.

## Methods

### Animals

All experiments were carried out in accordance with the Local Animal Care and Use Committee in Olsztyn, Poland (Agreements No. 76/2014/DTN and 55/2023). All experiments were conducted in accordance with relevant guidelines and regulations and adheres to the ARRIVE guidelines. The experiment took place in the owned by the University of Warmia and Mazuria in Olsztyn, Scientific and Educational Station in Bałdy. The animals used in the study resided at the Scientific and Educational Station in Bałdy, which belongs to the Warmia and Mazuria University in Olsztyn. During the experiment, the heifers stayed in their habitat and their environmental conditions did not change. All cattle were healthy and disease-free and were maintained under the same standard management conditions with free access to feed and water. Two groups of Polish Holstein-Friesian heifers were examined. The animals in the prepubertal group were younger than 10-months-old with the absence of a dominant follicle or corpus luteum in the ovary, which was confirmed by ultrasound examination. In the pubertal group, animals were older than 15-months-old and were confirmed by ultrasound examination to have a corpus luteum or dominant follicle on the ovary and were used as a control group.

## Chemicals and suppliers

All culture media for the in vitro production of bovine embryos were purchased from Minitube (Germany). From Nunc (Thermo Scientific Denmark), we received plastic dishes, 4-well plates, and tubes. Unspecific reagents and supplements for in vitro culture were obtained from Merck (Germany). The chemicals for reverse transcription were purchased from Invitrogen (Carlsbad, CA, USA). The SMARTer Stranded Total RNA-Seq Kit v3 - Pico Input Mammalian Components (cat. no. 634,489; Takara Bio Inc) was purchased to perform next-generation sequencing.

### Experimental design

The transcriptomic profiles of genes involved in oxidative phosphorylation pathway from blastocysts derived from oocytes collected from prepubertal and pubertal heifers were compared. TUNEL assay was performed to detect apoptotic cells in situ in blastocysts derived from oocytes collected from prepubertal and pubertal heifers.

### Ovum pick -up

Bovine cumulus oocyte complexes (COCs) were collected from the follicles from two studied groups of animals, prepubertal and pubertal via a transvaginal ultrasound quided OPU method, according to Cavalieri et al. [22]. Before each procedure of the ovum pick up, the perineal region was cleaned using water and 70% ethanol. The animals received standard epidural anesthesia before each OPU session with the use of polocainum hydrochloricum 2% and adrenalinum 0.005%. The dose of epidural anesthesia was determined based on the weight of the animal and ranged between 1,5 and 3 ml. Directly, after the experiment the animals came back to the herd and underwent normal reproductive management, as the rest of the animals leading to pregnancy at the age of approximately 13–16 months. None of the animals used for the study was sacrificed. The COCs were obtained in the same way as described in our previous study [21]. During the experiment fifty-one OPU sessions were performed. The oocytes collected by the OPU procedure were used for in vitro embryo production. COCs from researched groups were observed under an Olympus SZX10 stereomicroscope and washed twice in wash medium (TCM) supplemented with 20 mM HEPES, 25 mM sodium bicarbonate, 0.4% bovine serum albumin, and 40 µg/mL gentamicin. In vitro maturation was performed using obtained COCs.

### In vitro maturation (IVM)

The obtained COCs were pooled and placed (grouped separately from prepubertal and pubertal heifers) into 4-well plates (#144,444) containing 500 µL of maturation medium (TCM 199 Maturation Medium (19,990/0010), supplemented with 0.02 IU/mL of pregnant mare serum gonadotropin (PMSG, #G4527), 0.01 IU/mL human chorionic gonadotropin (hCG, #C0684,), and 5% fetal bovine serum (FBS, #12,106 C) and incubated at 38.5 °C in a 5% CO_2_ humidified atmosphere for 23 h for in vitro maturation (IVM).

### In vitro fertilization (IVF)

In all experiments frozen commercially available semen from the same bull (Sexing Technology, USA) was used for the IVF procedure. Computer- assisted sperm analysis method was used to assess and confirm the fertility parameters of the semen. The sperm were placed under capacitation medium [(TL sperm capacitation medium (19,990/0020) supplemented with 1 mM sodium pyruvate sodium pyruvate, 0.6% BSA and 0.1 mg/ml gentamicin)] after thawing in a water bath at 38 °C for 60 s, then incubated for one hour at 38.5 °C in a humidified atmosphere with 5% CO_2_ to allow recovery of motile spermatozoa using a swim-up procedure. Following incubation, the upper of two thirds of the capacitation medium were recovered and then centrifuged at 200 g for 10 min. Finally, the supernatant was removed and the sperm pellet was diluted in a appropriate volume of fertilisation medium to obtain the final concentration of 10^6^ motile sperm/mL. Groups of COCs, obtained from prepubertal and pubertal heifers were placed in 500 µl of fertilisation medium [(TL fertilization medium; 19,990/0030) supplemented with 10 µg/mL of heparin (#H3393), 20 mM sodium pyruvate (#P3662), and 0.5% BSA)] and co-incubated with spermatozoa in 4-well dishes containing 500 µL of fertilization medium for 24 h at 38.5 °C in an atmosphere humified with 5% CO_2_. The day of in vitro fertilization was regarded as day 0.

#### In vitro culture (IVC)

Embryos (from oocytes collected from prepubertal and pubertal heifers) were separated from the cumulus cells and attached sperm, then were washed twice in wash medium at 24 h after IVF. Then, blastocysts (groups of 25) were cultured in 4-well dishes cointaining 500 µL of culture medium [(SOF; 198 synthetic oviduct fluid medium (19,990/0040)] supplemented with basal medium Eagle-amino acids using 10 µL/mL (#B6766), 20 µL/mL MEM (#M7145), 3.3 mM sodium pyruvate, and 5% fetal bovine serum in 500 µL of mineral oil (NidOil, Nidacon). The culture conditions were carried at 38.5 °C in an atmosphere of 5% CO_2_, 5% O_2_, and 95% N_2_ with high humidity until the blastocyst stage was achieved (day 7). An Olympus SZX10 stereomicroscope was used to evaluate the development rate and quality of embryos obtained from oocytes collected from prepubertal and pubertal animals by morphological examination at approximately 100× magnification according to the guidelines of the International Embryo Technology Society (IETS). Embryo grading was used to select the appropiate blastocysts for the current study. For further analysis we used only blastocysts assesed as grade 1 and 2 (according to IETS). For RNAseq analysis blastocysts from both groups were stored separately in Lysis Mix (Takara Bio Inc) at − 80 °C. For quantitative polymerase chain reaction (qPCR) blastocysts from the two examined groups were stored at − 80 °C in Extraction Buffer (KIT0204, Arcturus PicoPure RNA Isolation Kit Applied Biosystems, CA, USA).

### Isolation of RNA from blastocysts for NGS sequencing

Total RNA was isolated from blastocysts derived from oocytes collected from prepubertal and pubertal heifers, as described previously [21]. For each group (blastocysts derived from prepubertal and pubertal heifers oocytes), total RNA was isolated from four replicates at an average of 5 pooled blastocysts (*n* = 4 × 5), and 4 vs. 4 samples were finally obtained for RNA-seq. The quality and quantity of mRNA were detected with a NanoDrop spectrophotometer (ND200C; Fisher Scientific, Hampton, PA, USA). The concentration of analyzed samples ranged between 0.6 and 2 ng/µl, and the RIN coefficient was 5.5 to 7.

Moreover, the RNA quality was controlled with TapeStation2200 (Agilent, Santa Clara, CA, USA) and High Sensitivity RNA ScreenTape (Agilent, Santa Clara, CA, USA) according to the manufacturer’s instructions. Only RNA samples with RNA integrity values between 5.5 and 7 were used for further analysis.

### Library preparation and whole transcriptome sequencing

Whole transcriptome sequencing was carried out for blastocysts derived from oocytes collected from prepubertal and pubertal heifers. For the analyzed blastocysts, the cDNA libraries were prepared according to the SMARTer Stranded Total RNA Seq Kit v3- Pico Input Mammalian (Takara Bio, USA, Inc). The libraries were prepared from 250 pg of total RNA according to the protocol. The samples were ligated with different adaptors and amplified in 5 cycles due to manufacturer’s recommendations depend on RNA input (5 cycles for 0.25 ng-10ng regular RNA samples). The final RNA-seq library amplification was performed in 12 cycles according to manufacturer protocol and preceded by personal validation of optimal number of cycles dedicated to the input amount of material. The quality and quantity of the obtained libraries were measured with Qubit (Invitrogen, ThermoFisher Scientific), TapeStation2200 (Agilent, Santa Clara, CA, USA) and High Sensitivity D1000 ScreenTape RNA ScreenTape (Agilent, Santa Clara, CA, USA) according to the manufacturer’s instructions. The concentrations of the libraries obtained were normalized to 10 nm, and the next samples were pooled. NGS sequencing was performed on a NextSeq500 Illumina platform in 75 single-end cycles according to the manufacturer’s instructions.

### Data analysis, DEGs identification and functional enrichment analysis

First, the raw reads were checked for quality using FastQC software, followed by a trimming procedure, which led to removing reads that did not meet the following criteria: <20 phred quality, reads shorter than 35 bp, and presence of adapters - adapters trimming (Flexbar) [23]. After the trimming procedure, the filtered reads were mapped to the Bos taurus UCD1.2 reference genome using STAR software [24].

In the next step, HTSeq-count software was used to count the mapped reads into the respective Ensembl GTF annotation file version 105. After assessing the read counts, DESeq2 software was used to calculate differentially expressed genes. The resulting genes with an adjusted *p* value < 0.05 and a fold change above > 1.2 were treated as differentially expressed for further analysis.

Differentially expressed genes in blastocysts obtained from oocytes collected from prepubertal and pubertal heifers were reported as gene lists in the DAVID v. 6.8 software [25] and KEGG databases (according to the *Bos Taurus* reference) and WebGestalt (WEB-based Gene SeT Analysis Toolkit [26] to confirm and perceive appreciably enriched Gene Ontology terms (GO) and pathways. KEGG enrichment analysis for differentially expressed genes was performed by http://www.bioinformatics.com.cn/srplot (Supplemental Figure S1).

### RNA isolation and qRT‒PCR for validation

Total RNA was isolated as described previously [21] from blastocysts obtained from prepubertal and pubertal heifers oocytes. RNA was extracted from (*n* = 4 × 5) blastocysts from prepubertal and (*n* = 4 × 5) blastocysts from pubertal heifers with an Arcturus PicoPure RNA Isolation Kit (#KIT0204, Applied Biosystems, USA) in accordance with the manufacturer’s instructions. First, SuperScript™ III First-Strand Synthesis SuperMix for qPCR (#11,752,250, Thermo Fisher, 278 USA) was used, and the products were stored at -20 °C.

### Validation of NGS data using real-time PCR (*n* = 4 × 5)

Complementary cDNA and qPCR analysis were performed using the same RNA from the prepubertal (*n* = 4 × 5) and pubertal (*n* = 4 × 5) blastocysts. The validation of RNA-seq results was designed using nine differentially expressed genes (DEGs). Genes were selected with an FC (> 1.2) and an adjusted *p* value < 0.05. Real-time PCR was used to check the expression of differentially expressed genes between the two examined groups (DEGs). The primers for real-time PCR were designed using the NCBI database (https://www.ncbi.nlm.nih.gov/tools/primer-blast/) and are presented in Table 1. Relative mRNA levels of genes used for validation were normalized to the *GAPDH* internal control. The obtained results were examined to be statistically significant for a *p* value < 0.05 using statistical analysis system software (GraphPad PRISM v. 9.0 Software, Inc., USA).

**Table 1:** Primers used for real-time PCR

Gene symbol	Gene name	Primer sequence 5′–3′	Amplicon size, bp	Gen Bank accession no.
***ATP5MF***	Bos taurus ATP synthase membrane subunit f	**F**: ACTGATGCGGGATTTCACCC **R**: TGCTCCCTTTCTTCACGTTCA	98	NM_001113719
***ATP5PD***	ATP synthase peripheral stalk subunit d	**F**: ACTTCCGTTCTCTGCTGCTGT **R**: CCCCAAAAGCTACCCAGTCAA	110	NM_174724.4
***ATP12A***	ATPase H+/K + transporting non-gastric alpha2 subunit	**F**: AAGCCTCGCCACAAGAAGAA **R**: CATGAGGCCGATATGCAGGT	78	XM_002691870.5
***NDUFA13***	NADH: ubiquinone oxidoreductase subunit A13	**F**: GCCCTATCGACTACAAGCGG **R**: TCCAGTACCCGAACAGCAAG	96	NM_176672
***CYCS***	cytochrome c, somatic	**F**: GCGTGTCCTTGGGCTTAGAA **R**: TTCTTTCTCTGTGCGCGACC	74	NM_001046061
***COX17***	cytochrome c oxidase copper chaperone COX17	**F**: GGACACCTAATTGAAGCCCAC **R**: ACCATGCTCACCATTTCATATCTT	72	NM_001207032.1
***UQCRC1***	ubiquinol cytochrome c reductase core protein 1	**F**: AGGACCTGCCAAAAGCTGTA **R**: CCCGCTCCTTCTCAATCTGG	84	NM_174629.2
***NDUFS3***	NADH: ubiquinone oxidoreductase core subunit S3	**F**: GGGAGGCTTTCCCTGCCTAT **R**: CCACATGCCTTCCCTGAAAC	99	NM_174819.3
***NDUFA3***	NADH: ubiquinone oxidoreductase subunit A3	**F**: GACCAAGATGGCTGAGAGAGT **R**: AATGGCGAAGGATGCCACTA	82	NM_176659
***GAPDH***	Glyceraldehyde 3-phosphate dehydrogenase	**F**: CACCCTCAAGATTGTCAGCA **R**: GGTCATAAGTCCCTCCACGA	103	NM_001034034.2

### Detection of apoptosis in blastocysts

For the detection of apoptotic cells in blastocysts derived from oocytes collected from prepubertal and pubertal heifers the terminal-uridine nick-end labeling (TUNEL) was conducted using the In Situ Cell Death Detection Kit, Fluorescein (#11,684,795,910, Roche, Germany). Blastocysts after culture from two examined groups were fixed in 4% paraformaldehyde in PBS for 1 h at room temperature (RT). Then, the blastocysts were permeabilized in 0.3% Triton X-100 (#T9284) in 0.1% sodium citrate for 2 min on ice and washed twice in PBS. To induce DNA strand breaks before TUNEL labelled positive control of blastocysts were treated with 3000 U/ml DNase (#79,254; Qiagen, Hilden, Germany) in the reaction buffer and incubated at RT for 10 min. Positive control and sample blastocysts were transferred to 50 µL of TUNEL enzyme (terminal deoxynucleotidyl transferase) reaction mixture and then incubated at 37 °C for 1 h in the dark. At the same time, negative control blastocysts were incubated in TUNEL label solution without the enzyme. After the incubation blastocysts were washed three times in PBS, and were stained with 10 µg/ml DAPI (#D9564) for 30 min at 30 °C in the dark. The blastocysts were observed under an LSM 800 confocal laser scanning microscope (Carl Zeiss, Germany) with a 40×/1.2NA immersion objective. The DAPI filter was used to estimate the total number of nuclei and the FITC filter to assess TUNEL positive cells.

### Assessment of apoptotic cells in blastocysts

The number of TUNEL positive cells per embryo was measured in two examined groups. To calculate the percentage of apoptotic cells a number of apoptotic cells divided by the total number of blastomeres per embryo. The experiment was conducted with 5 blastocysts per group. The percentage of apoptotic cells was calculated for each group. The obtained data were determined to be statistically significant for a *p* value < 0.05 using statistical analysis system software (GraphPad PRISM v. 9.0 Software, Inc., USA).

## Results

### Gene ontology analyses

RNA-Seq analysis was performed to compare the transcriptomic profiles of blastocysts derived from oocytes collected from prepubertal and pubertal heifers and to demonstrate the molecular mechanisms underlying the differences in the transcriptomic profiles.

The NGS statistic showed that the average raw reads obtained per sample were 53.9 mln, and 99% of them passed the quality filter (Table 2, Supplemental Figure S2). Moreover, an average of 79.4% of reads were uniquely mapped.

**Table 2:** The basic NGS statistic of all analyzed libraries

Sample	Raw reads	Filtered reads	Uniquely mapped	Mapped to multiple loci	Percent of uniquely mapped reads	Assigned to annotation database
1	52,571,938	52,051,424	41,581,031	6,363,643	79.88	24,828,302
2	54,708,852	54,167,180	41,703,329	6,705,325	76.99	24,164,077
3	56,123,912	55,568,230	44,273,299	6,886,066	79.67	20,112,044
4	57,650,297	57,079,502	46,430,720	7,192,302	81.34	29,549,143
5	53,208,628	52,681,810	43,023,223	6,788,176	81.66	27,266,940
6	57,591,774	57,021,558	45,434,202	8,041,295	79.67	28,098,361
7	43,469,751	43,039,357	35,545,624	4,607,069	82.58	18,510,164
8	56,320,387	55,762,759	41,149,301	8,518,737	73.79	24,831,086

**Fig. 1:**
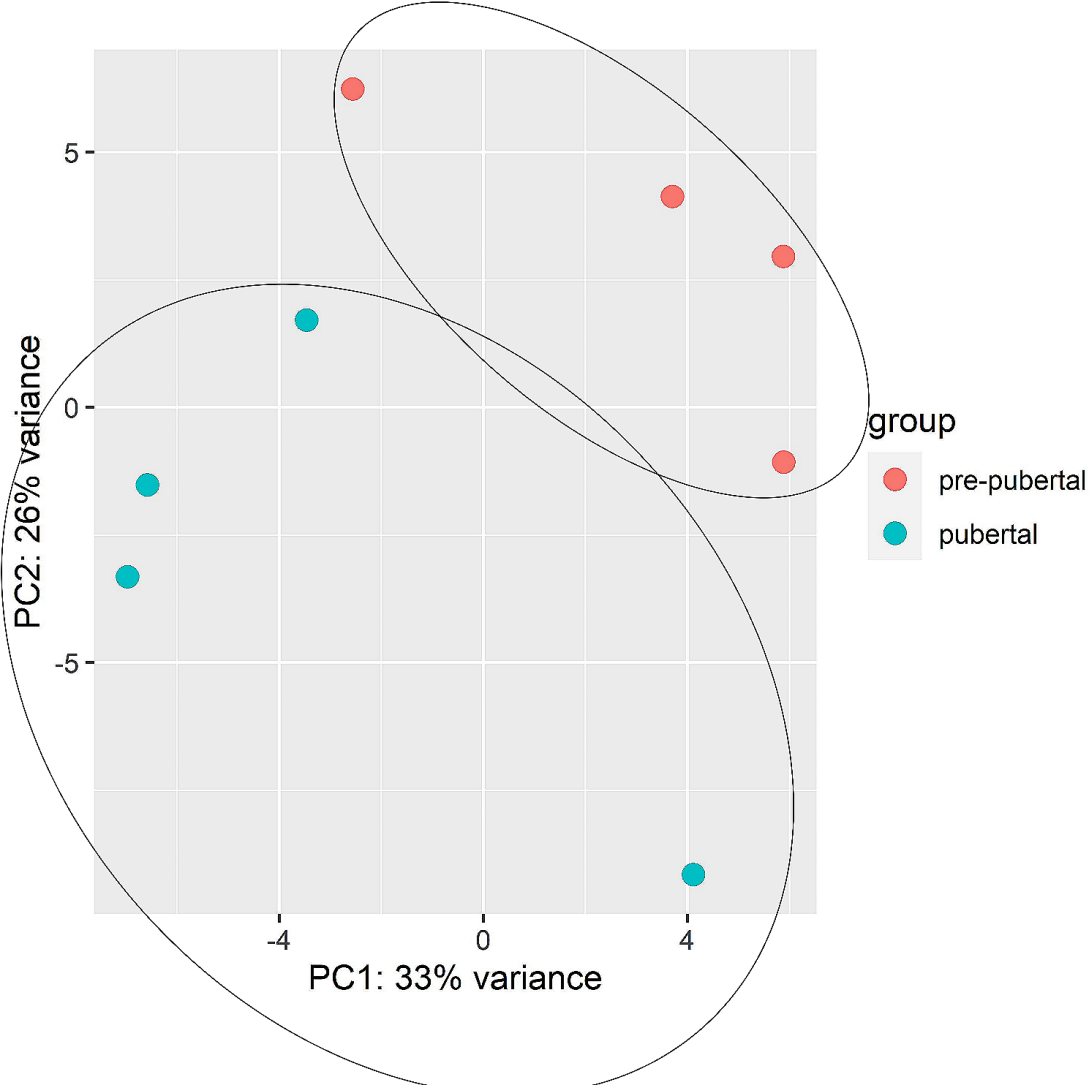
The Principal Component Analysis (PCA) clustering of samples representing two groups pre-pubertal (red) and pubertal (blue)

**Fig. 2:**
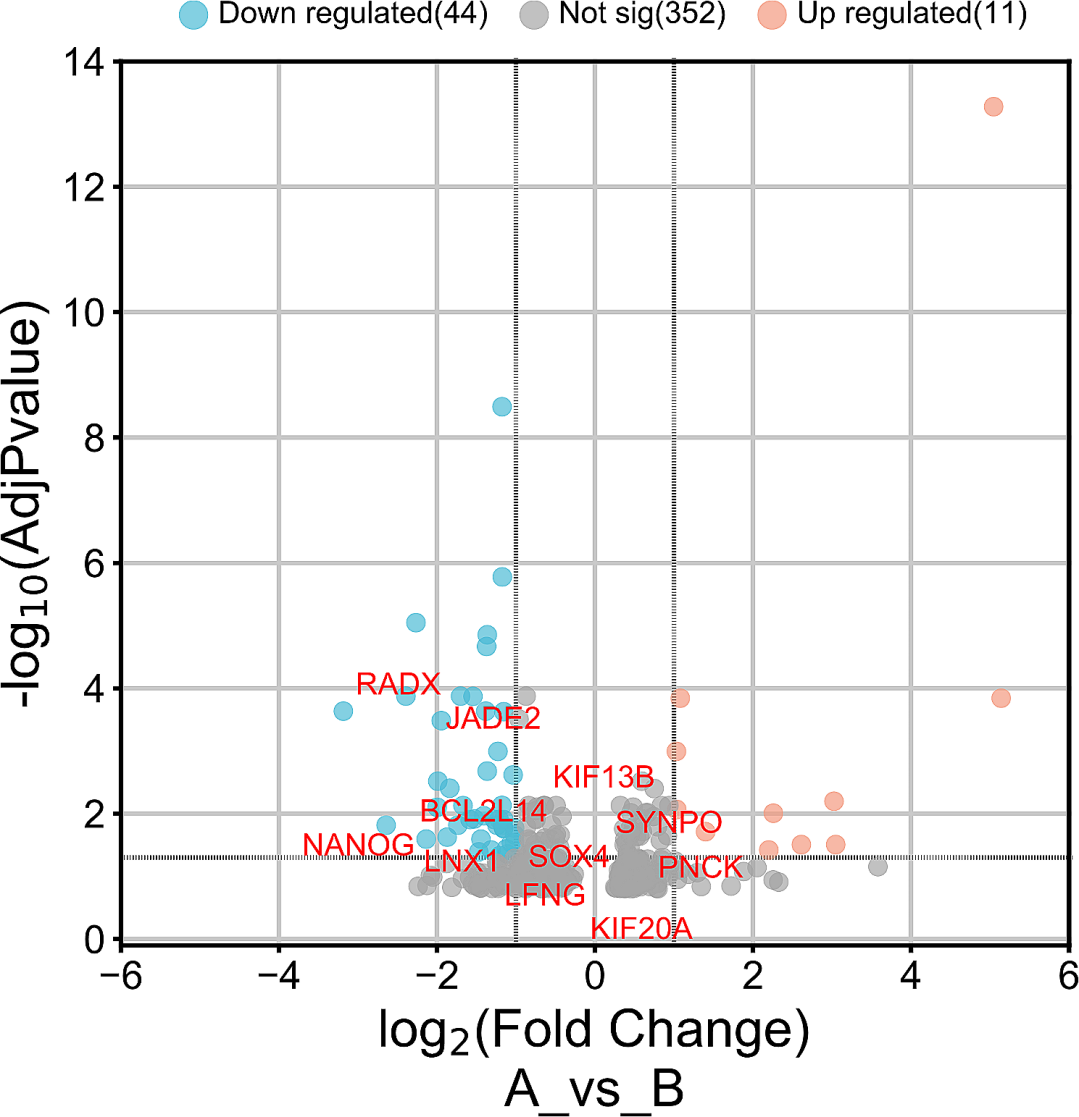
Volcano plot showing the significant up and down regulated genes across experiment designed. The DEGs belonged to oxidative phosphorylation pathway were red marked

We performed KEGG pathway enrichment analysis and found that mitochondrial OXPHOS was significantly overrepresented (FDR < 5.3E-1). We also presented significant interactions between analyzed differentially expressed genes (DEGs). The Principal Component Analysis (PCA) clustering of samples representing two groups pre-pubertal (red) and pubertal (blue) was presented in the Fig. 1. The volcano plot depicted the up and down- regulated DEGs in the experiment and was presented in Fig. 2. The DEGs belonged to oxidative phosphorylation pathway were red marked.

### Developmental competence of blastocyst stage and quality of the in vitro-produced blastocysts

The average number of oocytes per animal obtained from the pubertal group compared to the prepubertal group did not differ (*p* > 0.05). As shown in Table 3, the blastocyst rate was higher in the pubertal group than in the prepubertal group (*p* < 0.05). All collected blastocysts were graded (Grades 1–4) morphologically based on IETS criteria. Number and percentage of blastocysts in the different grades (Grades 1–4) are showed in Table 3. There were no differences in the quality of the obtained blastocysts between the two examined groups (*p* > 0.05). The total of 40 blastocysts Grade 1 were derived from prepubertal heifers oocytes and the total of 77 blastocysts were obtained from pubertal heifers oocytes. The percentage of Grade 1 embryos derived from oocytes collected from prepubertal heifers was higher than from the pubertal heifers, however this difference was not statistically significant. The percentage of Grade 2 blastocysts derived from oocytes collected from pubertal heifers was higher than that from pubertal, but not significantly different. The percentage of Grade 3 and 4 blastocysts were found to be similar in both groups and were no statistically different.

**Table 3:** Developmental rates of embryos

Experimental group	Total number of oocytes (*n*)	Average number of oocytes (*n*) per one animal	Total number of blastocyst (*n*)	Blastocyst rate (%)	Total number of selected quality blastocysts (%)
pre- pubertal	365	7.16	50	13.6^a^	Grade 1/40 [80]^a^ Grade 2/5 [10]^a^ Grade 3/4 [8]^a^ Grade 4/1 [2]^a^
pubertal	347	6.8	115	33.1^b^	Grade 1/77 [67]^a^ Grade 2/30 [26]^a^ Grade 3/5 [4]^a^ Grade 4/3 [3]^a^

**Fig. 3:**
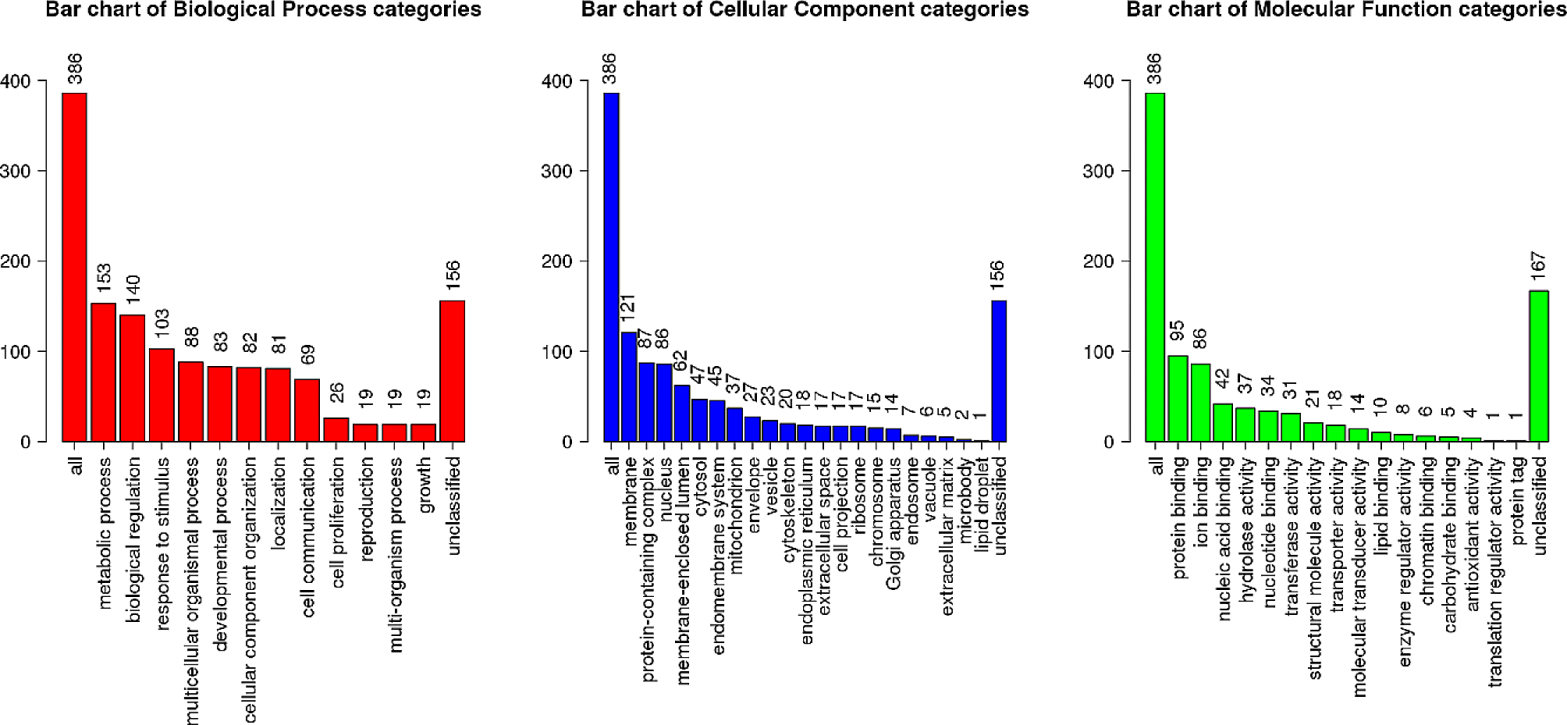
WebGestalt Analysis of the different Gene Ontology terms. Bar chart showing the number of genes from the nex-generation sequencing that are involved in the different Gene Ontology terms as predicted by the Gene Set Enrichment Analysis (GSEA) via WebGestalt. The graph is showing the number of genes involved in the different biological processes (Red), Cellular components (Blue) and Molecular functions (Green)

### Sequencing data Summary

#### Identification of differentially expressed genes (DEGs)

RNA-Seq of bovine blastocysts derived from oocytes collected from prepubertal and pubertal heifers was performed to identify, characterize and compare their transcriptomic profiles. Comparison of transcriptomic profiles showed that there were 436 differentially expressed genes between the two examined groups.

We noticed that 247 genes were downregulated in blastocysts derived from pubertal heifers compared to prepubertal heifers oocytes, and 189 differentially expressed genes were upregulated in blastocysts derived from pubertal heifers compared to prepubertal heifers oocytes. In the metabolic pathway 29 genes were up-regulated and 23 were down-regulated in embryos derived from pubertal compared to pre- pubertal heifers oocytes (Supplemental Figure S3, Supplemental Figure S4, Supplemental Table S1). In the oxidative phosphorylation pathway 8 genes were up-regulated and 1 was down-regulated in blastocysts derived from pubertal compared to prepubertal heifers oocytes (Supplemental Figure S5, Supplemental Figure S6, Supplemental Table S2).

#### Gene ontology analysis

Gene Ontology (GO) analysis was performed by DAVID (Database for Annotation, Visualization and Integrated Discovery) and WebGestalt [26]. Three categories from gene ontology were determined: biological process (BP), cellular component (CC) and molecular function (MF). Thirty-eight biological processes were found to be different in blastocysts derived from oocytes collected from prepubertal and pubertal heifers. A higher number of differentially expressed genes between the two examined groups was found in cell differentiation (GO: 0030154, 16 DEGs). The least number of differentially expressed genes were specified in negative regulation of primary miRNA processing (GO: 2,000,635, 2 DEGs), positive regulation of sodium ion transmembrane transporter activity (GO: 2,000,651, 2 DEGs), positive regulation of calcium- transporting ATPase activity (GO: 1,901,896, 2 DEGs), negative regulation of sodium ion transport (GO: 0010766, 2 DEGs), retina vasculature morphogenesis in camera- type eye (GO: 0061299, 2 DEGs) and mitral valvae morphogenesis (GO: 0003183, 2 DEGs).

Differentially expressed genes were involved in twenty-three processes in cellular component and twenty-six involved in molecular function.

We observed that several genes involved in oxidative phosphorylation were also related to other biological processes. Thus, the *CYCS* gene is also involved in the apoptotic process (GO: 0006915, 12 DEGs), *COX17* in mitochondrial respiratory chain complex IV (GO: 0033617, 3 DEGs) and *NDUFA13* in positive regulation in cystein-type endopeptidase activity involved in the apoptotic process (GO:0043280, 4 DEGs).

The analysis of differentially expressed genes distinguished from the three categories is shown in Fig. 3. designed by WebGestalt [26].

#### Pathway enrichment analysis

The identified DEGs were enriched in thirteen biological pathways, which was established by the KEGG (Kyoto Encyclopedia of Genes) tool (Table 4, Supplemental Figure S7, Supplemental Table S3). The most differentially expressed genes were observed in metabolic pathways (13 DEGs). The other biological pathways significant by DAVID were as follows: ribosome (13 DEGs), biosynthesis of cofactors (10 DEGs), glutathione metabolism (6 DEGs), oxidative phosphorylation (9 DEGs), carbon metabolism (7 DEGs), hypertrophic cardiomyopathy (6 DEGs), glycine, serine and threonine metabolism (4 DEGs), and microRNAs in cancer (11 DEGs).

**Table 4:** Analysis of pathways included DEGs^1^

Term	Accession number	Gene number	*p*-Value	Genes involved in the process
Ribosome	bta03010:Ribosome	13 (13- DEGs up-regulated)	2,2E-4	*RPL5, MRPL18, RPL13A, RPS3A, MRPL34, MRPL10, MRPL4, RPS25, MRPL2, RPS17, RPL14, RPL36, RPL27*
Metabolic pathways	bta01100:Metabolic pathways	52 (29- DEGs up-regulated, 23-DEGs down-regulated)	1,3E-3	*NDUFA13, ALAS2, ST6GALNAC2, ENO1, ATP12A, GPHN, GCSH, STS, ALDH2, ANPEP, CA4, ACP5, CD38, KMT5A, PSPH, PCYT1B, GPX3, MARS1, ITPK1, AMT, PGD, NME1, ALDH1A3, UQCRC1, NDUFS3, ALDOC, AGPAT5, DNMT1, ISYNA1, MGST3, COX17, MGST2, PTGS2, SELENBP1, GULO, DGUOK, UGP2, GMDS, RDH12, INPP5F, PNPO, ARSB, ATP5MF, PCK2, ATP5PD, NDUFA3, IDH2, ETNPPL, DHODH, MGAT4B, ALPL, CYCS*
Biosynthesis of cofactors	bta01240:Biosynthesis of cofactors	10 (5- DEGs up-regulated, 5-DEGs down-regulated)	4,9E-3	*GULO, ALAS2, UGP2, ALDH2, RDH12, PNPO, ALPL, GPHN, DHODH, NME1*
Glutathione metabolism	bta00480:Glutathione metabolism	6 (1- DEGs up-regulated, 5-DEGs down-regulated)	9,8E-3	*GPX3, MGST3, ANPEP, IDH2, MGST2, PGD*
Oxidative phosphorylation	bta00190:Oxidative phosphorylation	9 (8-DEGs up-regulated, 1-DEGs down-regulated)	1,1E-2	*NDUFA13, ATP5PD, NDUFA3, COX17, NDUFS3, UQCRC1, CYCS, ATP12A, ATP5MF*
Carbon metabolism	bta01200:Carbon metabolism	7(4-DEGs up-regulated, 3-DEGs down-regulated)	3,1E-2	*GCSH, IDH2, AMT, ALDOC, ENO1, PGD, PSPH*
Hypertrophic cardiomyopathy	bta05410:Hypertrophic cardiomyopathy	6(4-DEGs up-regulated, 2-DEGs down-regulated)	4,5E-2	*MYBPC3, RYR2, IL6, TPM3, TPM1, ITGB8*
Glycine, serine and threonine metabolism	bta00260:Glycine, serine and threonine metabolism	4(3-DEGs up-regulated, 1-DEGs down-regulated)	6,9E-2	*GCSH, ALAS2, AMT, PSPH*
MicroRNAs in cancer	bta05206:MicroRNAs in cancer	11(6-DEGs up-regulated, 5-DEGs down-regulated)	9,2E-2	*NOTCH3, ZEB2, DNMT1, TNN, STMN1, STAT3, TPM1, PTGS2, SLC7A1, SOX4, CDC25A*
Pathways of neurodegeneration- multiple diseases	bta05022: Pathways of neurodegeneration- multiple diseases	16(9-DEGs up-regulated, 7-DEGs down-regulated)	9,5E-2	*ATP5PD, NDUFS3, NDUFA13, NDUFA3, BDNF, CYCS, GPX1, IL6, NEFH. PTGS2, RYR2, TUBA4A, TUBB4A, TUBA1A, UQCRC1, UBE2G2*

^1^ The analysis was performed in two examined groups (embryos from prepubertal and pubertal heifers) according to DAVID database and demonstrated up and down regulated genes

#### Oxidative phosphorylation pathway

To demonstrate the importance of mitochondrial function in the oxidative phosphorylation pathway in blastocysts derived from prepubertal and pubertal heifers oocytes, we chose the oxidative phosphorylation pathway.

**Fig. 4:**
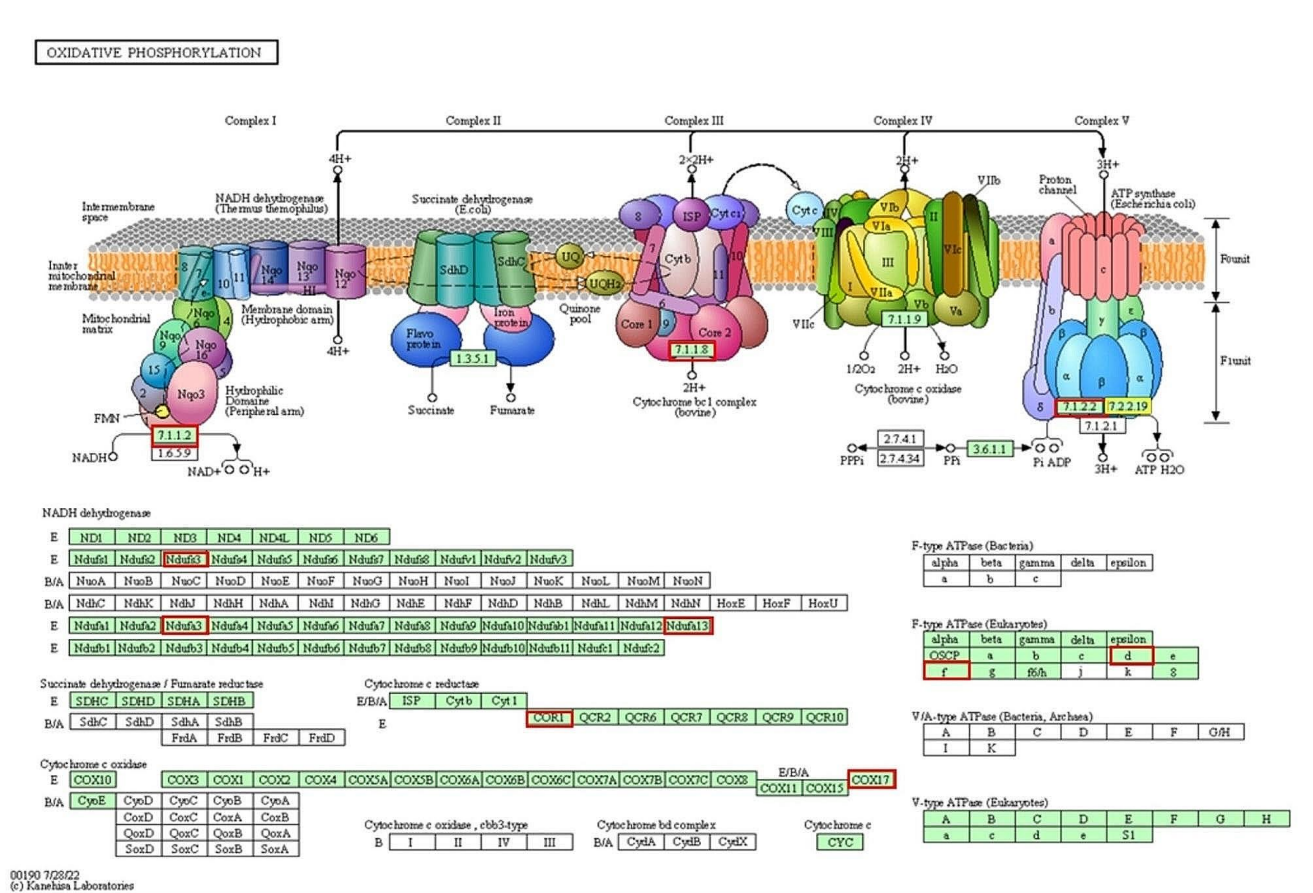
KEGG pathway diagram for oxidative phosphorylation. The functional pattern of the gene expression between pre- pubertal and pubertal groups. Boxes with red borders represent up- regulated genes and boxes with the yellow border represent down-regulated genes when the pre- pubertal group was compared with the pubertal group

The expression of eight genes involved in the oxidative phosphorylation pathway was upregulated in blastocysts derived from pubertal heifers compared to prepubertal heifers (Fig. 3, Supplemental Figure S6, Supplemental Figure S7, Supplemental Table S2). One of the genes associated with this pathway was downregulated in blastocysts derived from pubertal heifers compared to prepubertal heifers (Fig. 4).

#### Network between differentially expressed genes

The interaction network was constructed by STRING and Cytoscape (Supplemental Figure S8).

### qPCR validation of DEGs that were enriched in KEGG pathways

**Fig. 5:**
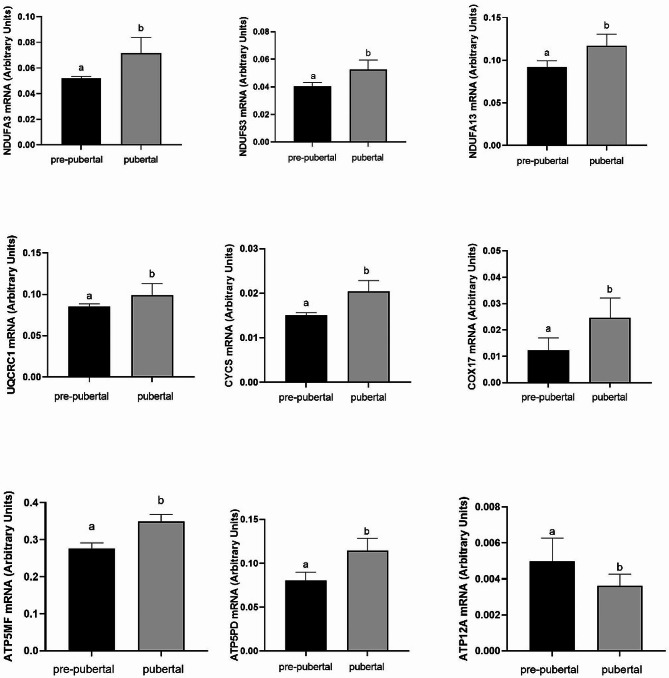
Real-time PCR validation in in vitro obtained bovine blastocysts (*n* = 4 × 5 for pre- pubertal and *n* = 4 × 5 for pubertal group) obtained from pre- pubertal and pubertal oocytes). Different letters above the column mean a significant differences between groups (*p* < 0.05), as determined by Student’s test. The data as presented as arbitrary units and are expressed as the mean ± SEM

We used real-time PCR to confirm the expression of nine DEGs involved in oxidative phosphorylation that were distinguished in blastocysts derived from prepubertal and pubertal heifers. Genes were selected with the FC (> 1.2) and adjusted *p* value < 0.05. The expression of *ATP5MF, ATP5PD, CYCS, COX17, UQCRC1, NDUFA3, NDUFA13* and *NDUFS3* was upregulated in blastocysts derived from pubertal compared to prepubertal heifers. The expression of *ATP12A* was downregulated in blastocysts derived from prepubertal heifers compared to pubertal heifers. The qPCR method showed that the expression of the *ATP5MF, ATP5PD, ATP12A, CYCS, COX17, UQCRC1, NDUFA3, NDUFA13* and *NDUFS3* genes was in agreement with the NGS analysis (Fig. 5). The obtained results confirmed the precision of highly developed RNA-Seq analysis.

### TUNEL assay

**Fig. 6:**
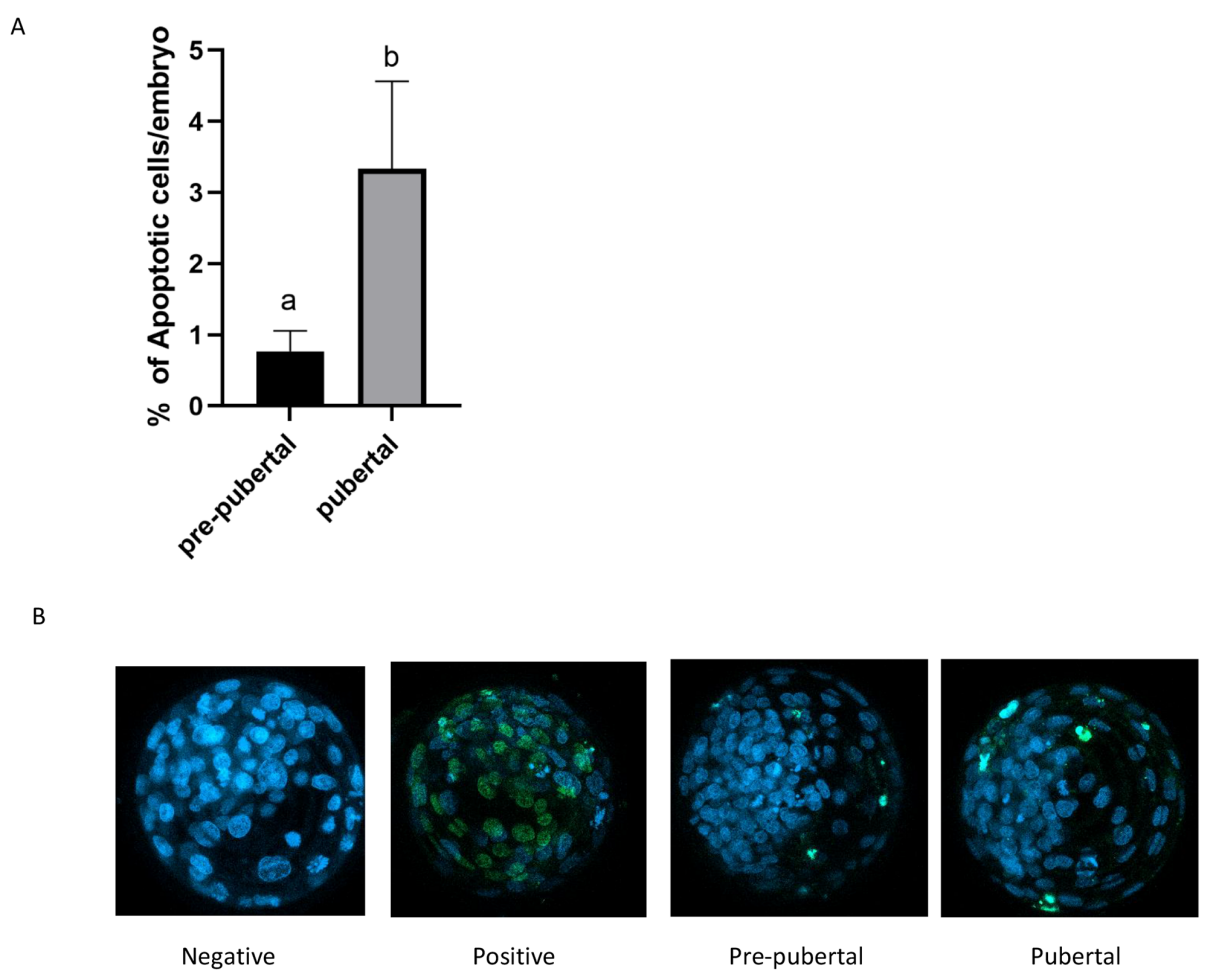
(**A**) TUNEL was used to asses the level of apoptosis in blastocysts derived from oocytes obtained from pre-pubertal and pubertal heifers. The number of individual cells that were TUNEL positive was counted in each blastocyst and is represented as the average number of cells that are TUNEL positive per blastocyst. Embryos derived from oocytes collected from pre-pubertal heifers displayed significant decrease the average number of TUNEL positive cells compared with blastocysts derived from oocytes obtained from pubertal heifers; mean ± SEM, *P* < 0.05 (**B**) Representative images of embryos from TUNEL assay. Negative: No fluorescence control; Positive: Embryos pretreated with DNAse to ensure TUNEL staining was successful; 5 embryos in each group

Blastocysts derived from prepubertal and pubertal heifers oocytes were assessed for apoptosis. Figure 6 depicts representative fluorescent images of bovine blastocysts labeled by TUNEL assay. The number of apoptotic cells was significantly higher in blastocysts derived from oocytes collected from pubertal heifers than those from prepubertal (*p* < 0.05) (Fig. 6).

## Discussion

The presented study showed that oocytes derived from prepubertal heifers had lower blastocyst rate than that obtained from pubertal heifers. Accurate knowledge about the transcriptomic profiles will enable us to gain insight into the molecular mechanisms involved in early embryonic development as well as early-fading fertility in cattle [27–29]. Morin-Dore et al. [30] demonstrated the analysis of transcriptome profile of blastocysts from prepubertal and pubertal heifers using microarray data analysis and presented the differences between examined groups and on the contrary with our study identified pathways such as mTOR (mammalian target of rapamycin), the peroxisome proliferator-activated receptor (PPAR) and NRF2-mediated oxidase stress response pathway. In the present study, we performed transcriptomic profile analysis of blastocysts derived from oocytes collected from prepubertal and pubertal heifers, but to the best of our knowledge for the first time demonstrated that the analysis of enriched KEGG pathways showed that DEGs were involved in the oxidative phosphorylation (OXPHOS) pathway, suggesting that this pathway was the most relevant in the conducted study and differed depending on the oocyte donor age. We have demonstrated that the expression of genes involved in OXPHOS had a crucial impact on embryo developmental competence, both in blastocysts derived from oocytes collected from prepubertal and pubertal heifers. Moreover, here, we also aimed to unravel the specific molecular markers of the embryos derived from prepubertal and pubertal heifers and their relationship with the developmental competence of examined embryos. In addition, identification of transcriptome profiles that favour early embryo development seems to be crucial for elucidating the genetic markers used to select developmentally competent embryos for embryo transfer.

In the conducted study, we identified 436 significantly differentially expressed genes, among which 247 genes were downregulated and 189 were upregulated when comparing the group of blastocysts derived from pubertal to the group of blastocysts derived from prepubertal heifers. Using the DAVID database, we identified that most of the differentially expressed genes were related to cellular components, biological processes and molecular function. Our study demonstrated that mitochondrial function exemplified by OXPHOS differed in blastocysts derived from oocytes obtained from prepubertal vs. pubertal heifers. Whereas mitochondria are significant organelles, described as powerhouses of cells, that are responsible for energy metabolism and ATP production [4], which in turn is associated with the growth, homeostasis or death of the cells [6, 27] the deregulation of their function might result in lower ATP production. It was previously demonstrated that the higher the ATP content in human and mouse MII oocytes, the better the embryo developmental and implantation ability [5]. Moreover, oocyte quality and embryo developmental ability depended directly on the better function of mitochondria expressed by significantly higher mtDNA copy number [31–33].

The OXPHOS pathway is a significant source of ATP production in mitochondria and depends on five multisubunit respiratory chain protein complexes that are directly involved in oocyte maturation [34], fertilization [35] and preimplantation development of embryos [36, 37]. In the current study, we found that the first and fifth complexes of the respiratory chain had the highest number of DEGs. The expression of nine genes (*NDUFA3, NDUFA13, NDUFS3, UQCRC1, ATP5MF, ATP5PD, ATP12A, CYCS, COX17*) involved in the OXPHOS pathway differed in blastocysts derived from oocytes collected from the pubertal group compared to the prepubertal group. The expression of *NDUFA3, NDUFA13, NDUFS3, ATP5MF, ATP5PD, CYCS* and *COX17* was higher in blastocysts derived from oocytes collected from pubertal heifers than in those derived from prepubertal heifers. Only the expression of *ATP12A* was higher in blastocysts derived from oocytes collected from prepubertal than pubertal heifers. Moreover, differences in oocyte competence derived from prepubertal heifers yield lower developmental competence compared to oocytes derived from pubertal heifers [38]. Our previous study mentioned that the expression of genes associated with developmental competence was lower in blastocysts derived from oocytes collected from prepubertal heifers than in those derived from pubertal heifers [21].

The largest complex in the respiratory chain of OXPHOS is complex I, called nicodine amide adenine dinucleotide [39–41]. This complex is considered a main source and entrance for electrons to the respiratory chain [42]. NADH, which is oxidized by NADH dehydrogenase, is produced in several processes, such as glycolysis, pyruvate dehydrogenase, the tricarboxylic acid (TCA) cycle, and beta-oxidation of fatty acids [43]. Furthermore, dysfunction of respiratory chain complex I is associated with many genetic diseases, including lactic acidosis, cardiomyopathy, leukoencephalopathy and muscle atrophy [44]. Therefore, the disorder in NADH dehydrogenase genes could cause dysfunction of the mitochondrial respiratory chain and a decrease in ATP production, as was demonstrated in blastocysts derived from prepubertal heifers. We found three differentially expressed genes, NADH: ubiquinone oxidoreductase subunit A3 (NDUFA3), NADH: ubiquinone oxidoreductase subunit A13 (NDUFA13), and NADH ubiquinone oxidoreductase core subunit S3 (NDUFS3), to be downregulated in blastocysts derived from oocytes collected from prepubertal compared to pubertal heifers, which might account for some dysfunctions of the mitochondrial respiratory chain action in those embryos. Moreover, NDUFA3, NDUFA13, and NDUFS3 have been previously considered to be predictive molecular markers of embryo developmental competence [1]. NADH: ubiquinone oxidoreductase subunit A3 and NADH: ubiquinone oxidoreductase subunit A13 are part of the ND1- module assembly, and NDUFA3 interacts with the transmembrane domain of NDUFA13. Moreover, NDUFA3 is located in the inner mitochondrial membrane and communicates with the previously mentioned core subunits ND1, ND3 and NDUFS8 and with the supernumerary subunits NDUFA8 and NDUFA13. Qin et al. [37] reported that the expression of NDUFA3 was downregulated in in vivo matured oocytes compared to in vitro matured oocytes, which are generally considered to have lower developmental competence [37]. In contrast, in our study, we demonstrated higher expression of NDUFA3 in blastocysts derived from oocytes collected from pubertal heifers than in those derived from prepubertal heifers, which in turn accounts for their better developmental competence.

Taking into consideration the higher expression of *NDUFA3, NDUFA13* and *NDUFS3* in pubertal heifers found in our study, we might presume that the blastocysts derived from heifers after they reach puberty have considerably higher developmental competence, caused by more efficient respiratory chain action. To support the hypothesis that lower expression of *NDUFA13* found in the blastocysts derived from oocytes collected from prepubertal heifers accounts for the probable higher lethality of those embryos, expressed by lower blastocyst rate in that group of blastocysts compared to the group from pubertal heifers, we would like to cite the studies of Huang et al. [45], Chao et al. [46] and Cui et al. [47] in mice. In those experiments, it was shown previously that the downregulation of the *NDUFA13* gene caused a reduction in mouse oocyte viability, maturation performance, and the developmental and implantation ability of embryos. Taking the above studies into consideration, we presume that the importance of NDUFA13 in bovine blastocysts obtained from oocytes collected from prepubertal and pubertal heifers is associated with the developmental competence of the embryos and suggest that the embryos derived from pubertal heifers had better developmental ability.

The other DEG we found in complex I of the respiratory chain is *NDUFS3*, which is the current precursor of mitochondrial assembly. Suhane et al. [48] indicated that the silencing of *NDUFS3* expression can systematically induce mitochondrial dysfunction. We found lower expression of *NDUFS3* in blastocysts derived from prepubertal heifers oocytes than in those derived from pubertal heifers oocytes. Zhang et al. [49] demonstrated significantly decreased expression of *NDUFS3* in Ca^2+^-overloaded oocytes and suggested its correlation with mitochondrial dysfunction in those oocytes [49]. The authors also mentioned that higher expression of *NDUFS3* could act as an ROS scavenger in those oocytes [49]. In our previous study [21], we also demonstrated higher ROS levels in blastocysts derived from oocytes collected from prepubertal heifers than in blastocysts derived from oocytes collected from pubertal animals. Furthermore [50], also suggested that higher Ca^2+^ levels in oocytes could induce mitochondrial oxidative stress and lead to apoptosis [50]. In our study, we might presume that the higher level of ROS, additionally associated with the lower expression of *NDUFS3* in blastocysts derived from oocytes collected from prepubertal heifers, might subsequently impair ROS scavenger efficiency in the blastocysts. Similarly, Sasaki et al. [51] showed that the higher expression of *NDUFS3* played a key role in reducing ROS levels and protected cells from cellular damage [51]. Zhang et al. [49] also demonstrated a negative correlation between ROS levels and the expression of the *NDUFS3* gene in murine oocytes [49]. Taking the obtained data into account, we presume that higher expression of the *NDUFS3* gene can act as an ROS scavenger in blastocysts derived from pubertal heifers as well as be a potential hypoxia marker associated with the status of the developmental competence of the embryo.

Another characteristic of embryos capable of surviving after transfer was the downregulation of genes associated with energy metabolism. Zolini et al. [52] indicated that 11 downregulated genes involved in oxidative phosphorylation were less expressed in blastocysts capable of surviving after transfer [52]. Similarly, other experiments conducted on embryos produced in vitro [53] and in vivo [54] confirmed this statement. In the studies carried out by El-Sayed et al. [53] and Ghanem et al. [54], embryos that resulted in calf delivery also had lower expression of two mitochondrial transcripts [53, 54]. Zolini et al. [52] also suggested that the downregulation of the *ND1* gene, which encodes one of the NADH dehydrogenase genes, could be one of the genes involved in oxidative phosphorylation, and its downregulation of expression facilitates a metabolically quiet phenotype [52]. Although we did not assess the expression of the *ND1* gene, we speculate that there is a predictable correlation in our study with lower expression of genes also associated with oxidative phosphorylation in blastocysts derived from prepubertal heifers and facilitating the metabolically quiet phenotype by those embryos.

The “quiet embryo” hypothesis was proposed by Leese and colleagues, according to which “thrifty” embryos that do not require high rates of metabolism have a better chance of sustaining successful development than embryos that are characterized by a high rate of metabolism [55, 56]. Additionally, we propose that the lower expression of genes involved in OXPHOS in blastocysts derived from oocytes collected from prepubertal heifers could be associated with the statement mentioned above. Such embryos would likely benefit from reduced oxygen consumption, energy and nutrient requirements [52]. However, some studies on embryo transfer have not confirmed this statement [52]. Considering the research on human embryos produced in vitro and their pregnancy establishment ability after transfer, the authors noted significantly higher use of glucose by those embryos [13]. Moreover, in the cow, significant differences were demonstrated in oxygen consumption by the blastocysts developed in vivo; nevertheless, there was a nonsignificant tendency for a higher pregnancy rate for blastocysts using higher oxygen [57].

Additionally, many gene clusters associated with OXPHOS were also found in the present study in blastocysts derived from oocytes collected from prepubertal vs. pubertal heifers. In complex V, we identified three differentially expressed genes, namely, *ATP5MF, ATP5PD* and *ATP12A*. *ATP5MF* (ATP synthase membrane subunit f) and *ATP5PD* (ATP synthase peripheral stalk subunit), that, as demonstrated by Fu et al. [58], could be considered molecular markers for embryo implantation [58]. The upregulation of *ATP5PD* in reactivated blastocysts compared to dormant blastocysts was demonstrated by Fu et al. [58], who subsequently suggested that ATP production was reinforced in the mitochondria during the reactivation of blastocysts [58]. Moreover, Salilew et al. [1] found higher expression of *ATP5PD* in competent compared to noncompetent in vivo-derived embryos, which is consistent with data obtained in our study. We noticed higher expression of the *ATP5MF* and *ATP5PD* genes in blastocysts derived from oocytes collected from pubertal heifers compared to prepubertal heifers, which in turn accounts for the better developmental competence and implantation ability of those embryos.

In our previous study, we documented that *ATP5F1A* (ATP synthase subunit F1 alpha, also known as ATP5A1) was also overexpressed in blastocysts derived from prepubertal compared to pubertal heifers [21]. Liu et al. [59] demonstrated that the decreasing expression of *ATP5A1* was correlated with the aging process and that this gene had an essential role in the disorder of the function of mitochondria in human granulosa cells of aging women [59].

Ubiquinol-cytochrome C reductase core protein I (UQCRC1), a mitochondrial protein, is known as a biomarker of Alzheimer’s disease and is located in complex III in the respiratory chain [60, 61]. UQCRC1 acts only as a core protein to simplify the interaction between cytochrome c (complex IV component) and cytochrome c1 (complex III component) [62]. The downregulation of *UQCRC1* expression may lead to decreased complex III activity, the consequence of which is the disruption of mitochondrial membrane potential as well as the reduced production of ATP with the increase in the level of ROS [63]. UQCRC1 plays an essential role in embryo survival and development [64]. Previous studies demonstrated that the knockout of *UQCRH* reinforced the Warburg effect [65]. This phenomenon has a crucial role in embryogenesis in the process of supporting rapid cell proliferation [66]. Qin et al. [37] demonstrated the downregulation of *UQCRC1* in in vitro matured oocytes from mice, which had higher levels of ROS than in vivo matured oocytes [37]. We presume that the lower expression of the *UQCRC1* gene in blastocysts derived from prepubertal heifers oocytes compared to pubertal heifers oocytes could also lead to a higher level of ROS, which we demonstrated in a previous study in those blastocysts [21], and at the same time reinforce the Warburg effect. We therefore assume that a higher level of that gene in blastocysts derived from pubertal heifers can be associated with better embryo developmental competence.

The inner- intermembrane electrochemical potential generated by OXPHOS in addition to ATP production is a significant feature of the organelle, which is useful for other important mitochondrial functions, such as dysfunction of mitochondria in response to protein import [67]. We investigated the expression of genes, such as *COX17* and *CYCS*, which are located in the mitochondrial intermembrane space and play a role in embryo developmental competence. Cytochrome c oxidase copper chaperone (COX17), also involved in OXPHOS, is located in the mitochondrial intermembrane space and is essential for the transport of copper to the mitochondria as well as cytochrome c oxidase activity [68], which is required for mitochondrial intermembrane space assembly and Cu homeostasis in cells [69]. In blastocysts, cytochrome c oxidase activity subunits were documented to be involved in embryo developmental competence and implantation ability [58]. Fu et al. [58] who investigated the global protein profiles in blastocysts at dormancy versus at reactivation, showed the downregulation of the *COX17* gene in reactivated blastocysts [58]. Similarly, in our study, we documented lower *COX17* expression in blastocysts derived from oocytes collected from prepubertal heifers compared to pubertal animals, which in turn accounts for the lower embryo developmental competence of those embryos. Moreover, Ntostis et al. [70] compared the transcriptomal profile of GV and MII oocytes from advanced maternal age (AMA) and young maternal age (YMA) groups [70]. Notably, those authors indicated that 31 genes involved in OXPHOS were highly expressed in young maternal age (YMA), such as cytochrome c oxidases (COX gene family), including the *COX17* gene, highlighting the significance of genes from that family in energy production and potential results in MII oocyte physiology and developmental capability [71]. In contrast, we propose that higher expression of *COX17* in blastocysts derived from pubertal heifers oocytes in our study could also be associated with energy production and have an impact on developmental competence in those blastocysts.

Cytochrome c somatic (CYCS), also located in the mitochondrial intermembrane space, is required for the electron transfer protein during OXPHOS, acting by transferring one electron from the cytochrome bc1 complex to cytochrome c oxidase [72], and its activity is essential for life [73]. Moreover, cytochrome c is also directly involved in apoptosis while being released into cytosol as a coactivator for caspase-3- activation as it binds the protease activating factor 1, leading to downstream activation of effector caspases [74–77]. It was previously demonstrated that the disruption of the unique *CYCS* gene caused embryonic lethality in mice [78]. We also demonstrated lower expression of the *CYCS* gene in blastocysts derived from prepubertal compared to pubertal heifers oocytes, suggesting that the lower expression of *CYCS* in blastocysts derived from prepubertal heifers oocytes was associated with lower developmental competence via modulation of the apoptotic process in the cells.

However, in our study, only one of the DEGs involved in OXPHOS, ATPase H+/K + transporting non gastric alpha2 subunit (*ATP12A*), which belongs to the cation transport ATPase (P-type), was demonstrated to have higher expression in blastocysts derived from oocytes collected from prepubertal heifers than in those derived from pubertal heifers. Qin et al. [37] demonstrated higher expression of *ATP12A* and ROS levels in in vitro matured (IVM) oocytes compared to in vivo matured (IVO) oocytes derived from mice [37]. We also indicated higher expression of *ATP12A* in blastocysts derived from oocytes collected from prepubertal heifers than in blastocysts derived from oocytes collected from pubertal animals, which correlated with previously demonstrated higher expression of ROS in those blastocysts, as confirmed by immunofluorescence staining [21]. Zhang et al. [42] also demonstrated that genes involved in OXPHOS were downregulated in GV-stage oocytes from 32-week-old mice in comparison with 5-week-old mice, which suggests that aging could have a detrimental impact on the mitochondrial respiratory chain in those oocytes [42]. Jakab et al. [79] suggested that in monocytes, higher expression of *ATP12A* could play a role in counteracting apoptosis via apoptotic volume decrease (AVD), intracellular acidification and a decrease in intracellular K^+^ [79]. Programmed cell death depends on changes in the intracellular ion composition and subsequently the volume of cells, associated directly with the impaired function of Na^+^, K^+^ ATPase, which in turn leads to the loss of K^+^ and gain of Na^+^ ions [80]. AVD and cell shrinkage are triggered by the activation of cell volume regulation connected with potassium levels and anion channels. These processes lead to the cellular exit of K^+^, Cl^−^ and HCO_3_^−^ ions and osmotically obligated water [81–83]. Intracellular acidosis associated with the intracellular loss of these ions is necessary for the execution of apoptosis. Therefore, the expression of *ATP12A* could be considered the gene for blunting intracellular acidosis and counteracting the loss of K + ions and AVD. As we demonstrated higher expression of *ATP12A* in blastocysts derived from oocytes collected from prepubertal compared to pubertal heifers, we presume that ATP12A is involved in counteracting the early stages of apoptosis and maintaining the growth of the prepubertal blastocyst. Therefore, higher expression of *ATP12A* could be considered a potential candidate to counteract the process of early apoptosis.

Apoptosis is a physiological process which occurs during embryonic development [84]. Several studies demonstrated that the association between the presence of apoptosis in oocytes and blastocysts associated with their developmental competence seen to be inconsistent [85–89] Study of Bilodeau-Goeseels [86] demonstrated that oocytes which are developmentally competent have early signs for atresia. However, increased apoptosis is often in association with the worse embryo development and may lead to arrest the embryonic development before compaction. The study of Zaraza [90] aimed to compare the apoptosis level in blastocysts derived from oocytes collected from prepubertal calves, postpubertal calves and adult cows and demonstrated the lower level of apoptotic cells in blastocysts from adult cows compared to both prepubertal and postpubertal animals, which is in contrast to our results.

However, in the conducted study we demonstrated lower level of apoptotic cells in embryos derived from oocytes collected from prepubertal heifers which in turn can be beneficial to reach the blastocyst stage leading to their better chance for survival after transfer as well as. Zaraza [90] compared the apoptotic level in blastocysts derived from prepubertal, postpubertal and adult cows and found that the most significant factor determining the apoptosis was the age of oocytes donor. Moreover, the excess of glucose or glucose deprivation could cause the apoptosis. Several studies [90–92] mentioned that the relationship between decreased level of GLUTs transporter is related to programmed cell death by triggerring the apoptotic cascade in blastocysts. With the agreement on the above statement, in our previous study [21] we demonstrated the higher level of glucose transporters such as SLC2A1 and SLC2A5 in blastocysts derived from oocytes collected from prepubertal heifers and in the current study the higher level of ATP12A cause the decreased level of apoptosis in those blastocysts. The lower expression of ATP12A could act as counteracting the early stages of apoptosis in blastocysts derived from oocytes collected from prepubertal heifers and facilitates a metabolically quiet phenotype. As Singh et al. [93] documented that ATP12A was a transcriptional marker of committed lineages in the human blastocysts, therefore we presume that higher expression of ATP12A in blastocysts derived from oocytes collected from prepubertal heifers in our experiments can account for better early embryo preimplantation development. Moreover, Bogliotti et al. [94] demonstrated transcripts associated with the maternal-to-embryo transition and using GO analysis of transcripts documented enrichment of ATP12A in MII eggs, not in 8–16 cell embryo in terms associated with ion transport and nucleotide biosynthetic process. Furthermore, in the study conducted by Sood et al. [95] demonstrated the downregulation of ATP12A in blastocysts derived from cloned than IVF group and mentioned that the genes involved in embryo development are uniqely expressed in IVF group. Considering above, we pressume that the higher expression of ATP12A in blastocysts derived from oocytes collected from prepubertal heifers counteract the early stages of apoptosis, but is associated with poor developmental competence in those embryos.

Moreover, in our study, to broaden our knowledge about the function of all the DEGs in the examined blastocysts, we analyzed the molecular pathways in which these clusters were involved. ATP production in blastocysts could occur via aerobic and anaerobic energy-providing pathways. Salilew-Wondim et al. [1] compared *in vivo-* and in vitro-derived embryos using NGS sequencing and previously documented that a higher ATP accumulation ability in more competent in vivo-derived embryos was directly associated with the ability to utilize both aerobic and anaerobic energy-providing pathways [1]. Thompson et al. [12] also documented long ago that 86% of ATP production in produced bovine blastocysts in vitro was obtained via OXPHOS, which in turn explains that aerobic pathways are the main pathways of ATP production at the blastocyst stage [12]. Similarly, we compared DEGs involved in OXPHOS in blastocysts derived from prepubertal and pubertal heifers oocytes and confirmed the findings of Salilew-Wondim et al. [1] and Thompson et al. [12] that ATP production is obtained through aerobic pathways. Zolini et al. [52] mentioned that the decreased expression of genes involved in OXPHOS was associated with the survival of in vivo-produced female blastocysts to Day 60 of pregnancy [52]. Similarly the lower expression of genes involved in OXPHOS in blastocysts derived from prepubertal heifers oocytes found in our study does not have to account for their lower implantation ability, which is reflected by lower number of apoptotic cells in those blastocysts. However to explicitly confirm our hypothesis further experiments concerning embryo transfer are needed. Moreover, gaining new knowledge about the transcriptome analysis in embryos derived from prepubertal and pubertal heifers oocytes will enable us to search for more comprehensive overview about the possibility for selecting the embryos for transfer. Moreover, created for this study new data base of the whole transcriptome profiles of blastocysts derived from prepubertal and pubertal heifers oocytes gives unlimited potential for searching new molecular candidates for selecting embryos for embryo transfer.

## Conclusions

In conclusion, the findings of our study provide new insight into the transcriptomic profile of blastocysts derived from prepubertal and pubertal heifers. We demonstrated differences in the expression of genes involved in mitochondrial function and OXPHOS in two groups of examined blastocysts, can be used for choosing the embryos with higher developmental competence for embryo transfer. So far, the selection of transferable embryos is mainly based on morphological criteria. Therefore, created for this study data base of the whole transcriptome profiles of blastocysts derived from prepubertal and pubertal heifers oocytes gives new possibilities for searching molecular markers of implantation ability. Moreover, the increased expression of ATP12A, together with the lower number of apoptotic cells in blastocysts derived from prepubertal heifers oocytes does not restrain their ability to produce full-term pregnancies. The possibility of using blastocysts derived from prepubertal heifers in embryo transfer programs will directly provide possibilities to enhance genetic gain in domestic livestock via the reduction of generation interval.

### Electronic supplementary material

Below is the link to the electronic supplementary material.

Supplementary Material 1

Supplementary Material 2

Supplementary Material 3

Supplementary Material 4

Supplementary Material 5

Supplementary Material 6

Supplementary Material 7

Supplementary Material 8

Supplementary Material 9

Supplementary Material 10

Supplementary Material 11

## Data Availability

The data have been submitted to the Gene Expression Omnibus (GEO) database and are available under GSE242449 accession number.

## Abbreviations

ATP: adenosine triphosphate
COCs: cumulus-oocyte complexes
DAVID: Database for Annotation, Visualization and Integrated Discovery
KEGG: Kyoto Encyclopedia of Genes
DEGs: differentially expressed genes
ATP5MF: ATP synthase membrane subunit f
ATP5PD: ATP synthase peripheral stalk subunit d
ATP12A: ATPase H+/K + transporting non-gastric alpha2 subunit
NDUFS3: NADH: ubiquinone oxidoreductase subunit core subunit S3
NDUFA13: NADH: ubiquinone oxidoreductase subunit A13
NDUFA3: NADH: ubiquinone oxidoreductase subunit A3
COX17: cytochrome c oxidase
CYCS: cytochrome c somatic
UQCRC1: ubiquinol cytochrome c reductase core protein 1
OXPHOS: oxidative phosphorylation
IVP: in vitro production
EGA: embryonic genome activation
OPU: ovum pick up
WebGestalt: WEB-based Gene SeT Analysis Toolkit
Rna-seq: RNA sequencing
GO: Gene Ontology
TUNEL: The terminal-uridine nick-end labeling
IVM: In vitro maturation
IVC: In vitro culture
IETS: International Embryo Technology Society
qPCR: quantitative polymerase chain reaction
