# Supplementary material for: Deregulation of oxidative phosphorylation pathways in embryos derived in vitro from prepubertal and pubertal heifers based on whole-transcriptome sequencing
Source: BMC Genomics. 2024 Jun 24;25:632. doi: 10.1186/s12864-024-10532-7 (PMC11197288; doi:10.1186/s12864-024-10532-7)
Supplement: Supplementary file 1 — Supplementary Material 1 [file 12864_2024_10532_MOESM1_ESM.docx]

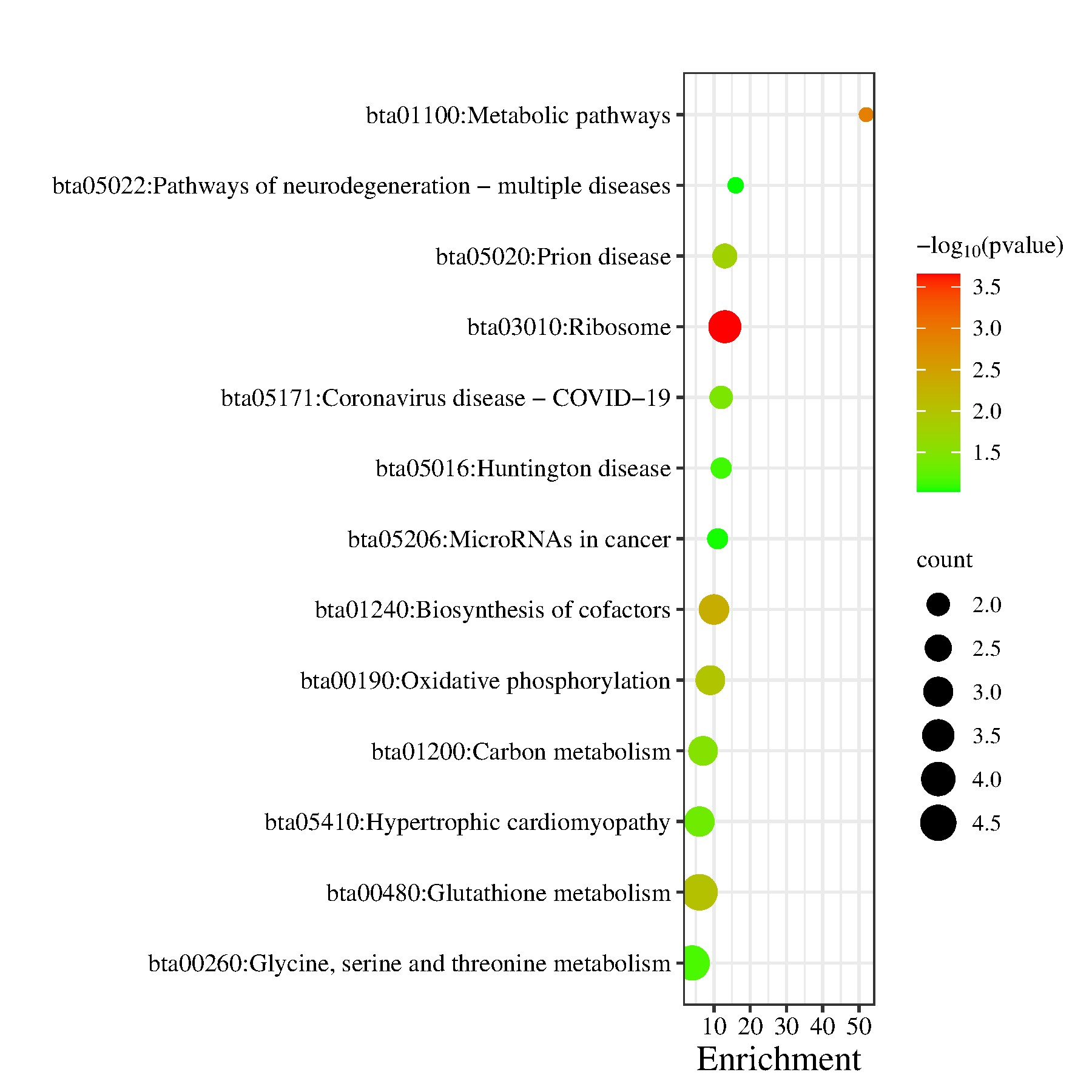


**Supplemental Figure S1.** KEGG pathway enrichment analysis. Y-axis indicate the pathway name, x-axis indicates the enriched factor in each of pathways. The bubble sizes are associated with the numer of DEGs. KEGG pathway enrichment analysis was perofrmed by <http://www.bioinformatics.com.cn/srplot>.
