# Supplementary material for: Deregulation of oxidative phosphorylation pathways in embryos derived in vitro from prepubertal and pubertal heifers based on whole-transcriptome sequencing
Source: BMC Genomics. 2024 Jun 24;25:632. doi: 10.1186/s12864-024-10532-7 (PMC11197288; doi:10.1186/s12864-024-10532-7)
Supplement: Supplementary file 2 — Supplementary Material 2 [file 12864_2024_10532_MOESM2_ESM.docx]

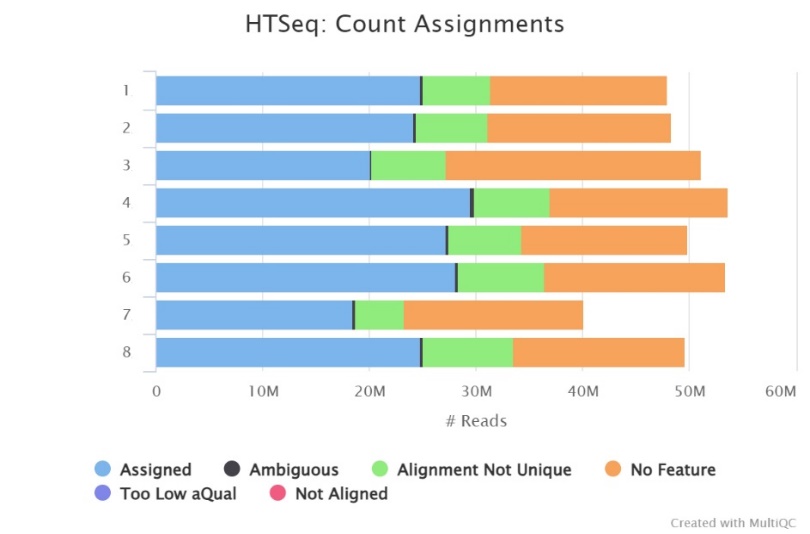


A


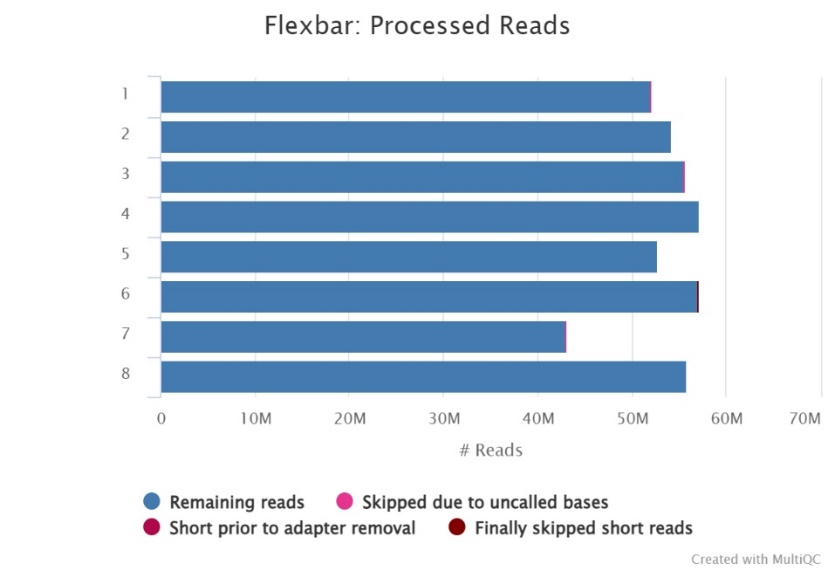


B


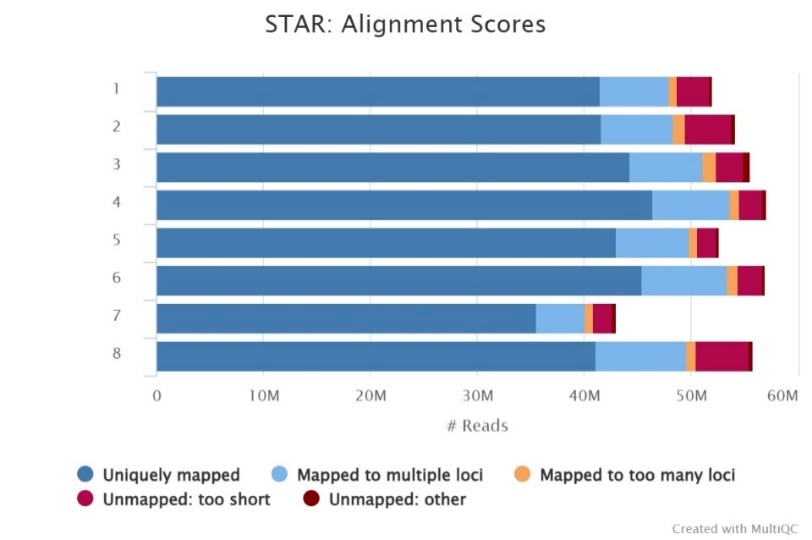


C

**Supplemental Figure S2.** (A). HTSeq Counts Assignments, (B) Flexbar: Processed reads, (C) STAR: Alignment Score.
