# Supplementary material for: Deregulation of oxidative phosphorylation pathways in embryos derived in vitro from prepubertal and pubertal heifers based on whole-transcriptome sequencing
Source: BMC Genomics. 2024 Jun 24;25:632. doi: 10.1186/s12864-024-10532-7 (PMC11197288; doi:10.1186/s12864-024-10532-7)
Supplement: Supplementary file 3 — Supplementary Material 3 [file 12864_2024_10532_MOESM3_ESM.docx]

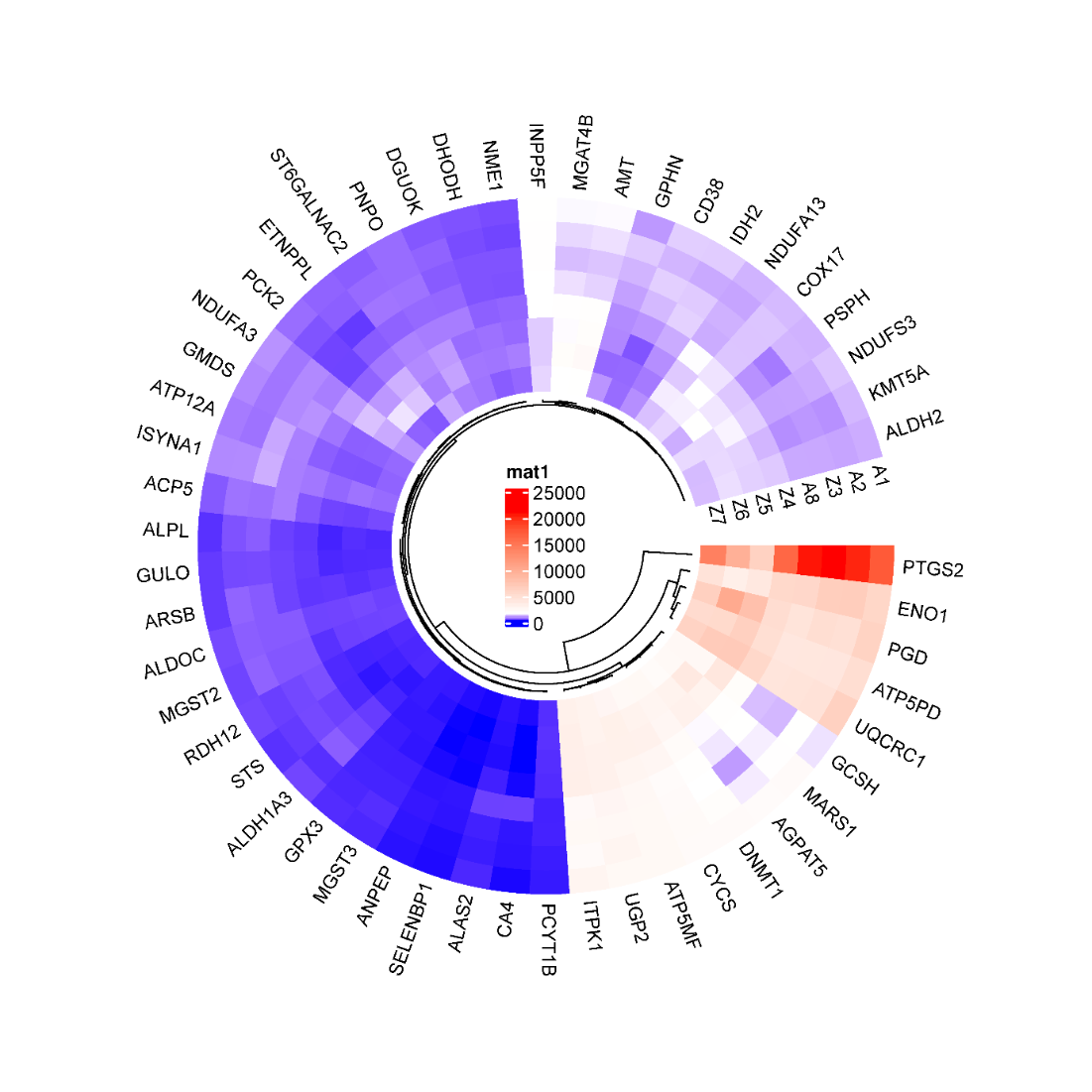


**Supplemental Figure S3.** The heatmap corresponding to the identified DEGs in metabolic pathways.
