# Supplementary material for: Deregulation of oxidative phosphorylation pathways in embryos derived in vitro from prepubertal and pubertal heifers based on whole-transcriptome sequencing
Source: BMC Genomics. 2024 Jun 24;25:632. doi: 10.1186/s12864-024-10532-7 (PMC11197288; doi:10.1186/s12864-024-10532-7)
Supplement: Supplementary file 7 — Supplementary Material 7 [file 12864_2024_10532_MOESM7_ESM.docx]

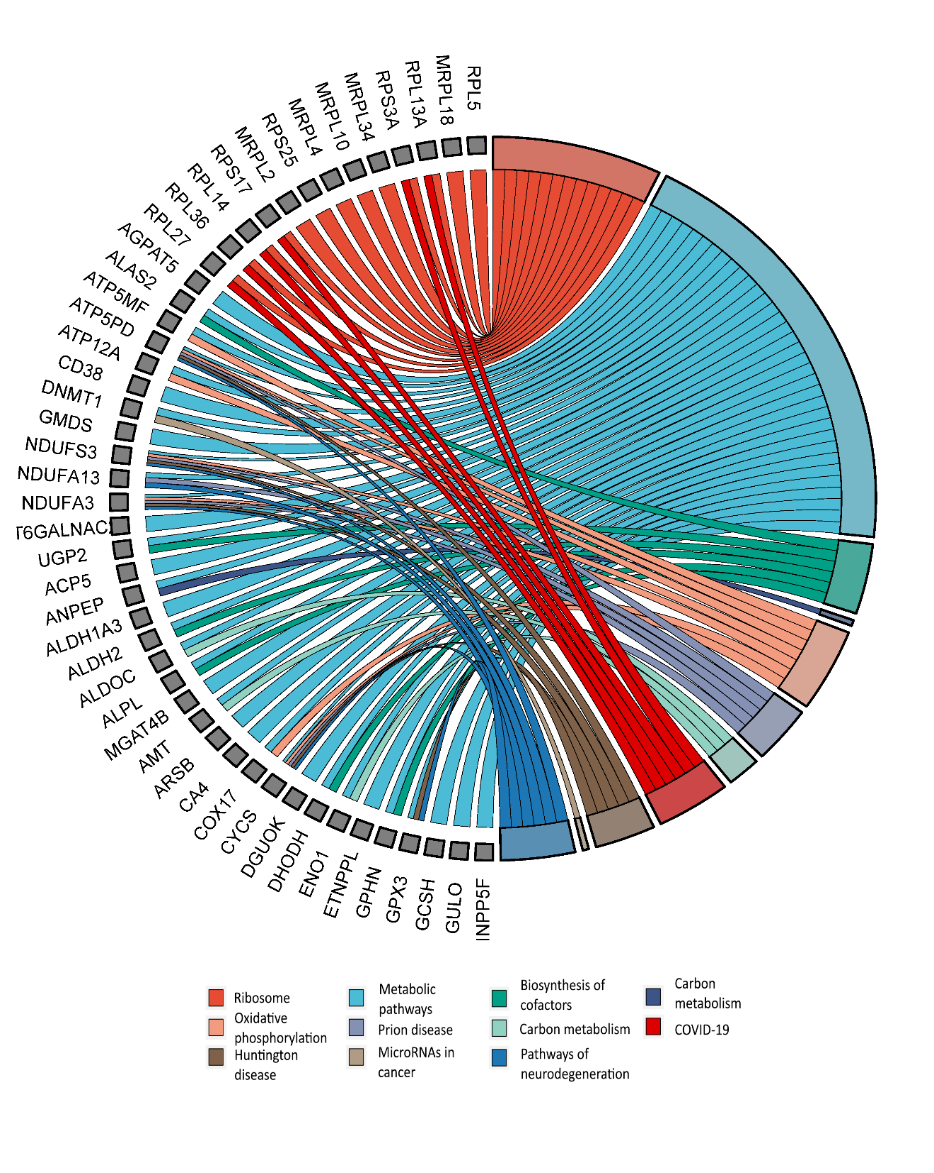


**Supplemental Figure S7.** The chord diagram represents DEGs associated with the enriched KEGG pathways (Ribosome, Oxidative phosphorylation, Huntington disease, metabolic pathways, prion disease, MicroRnas in cancer, Biosynthesis of cofactors, Carbon metabolism, Pathways of neurodegeneration, Additional File 7. Carbon metabolism, COVID-19.
